# Effect of Insurance Subsidies on Agricultural Land-Use

**DOI:** 10.3390/ijerph20021493

**Published:** 2023-01-13

**Authors:** Chengyu Si, Yanru Li, Wei Jiang

**Affiliations:** 1 School of Economics, Hangzhou Normal University, Hangzhou 311121, China; 2 Nanjing Intellectual Property Protection Center, Nanjing 211800, China

**Keywords:** land use, insurance subsidies, multinomial- fractional-logit model

## Abstract

This paper investigates the effect of crop insurance-subsidies on agricultural land-use allocation. Since the objective of crop insurance is to help farmers with risk management, the expected profit from crop production under crop insurance might be improved, leading farmers to allocate more land to crop production. In this paper, agricultural land-use type is classified by irrigated/unirrigated farmland and cropland/woodland/pastureland. The data come from counties from all the continental states. Considering the fractional outcome of land-use share, we apply a multinomial-fractional-logit model to estimate the effects. The results show that insurance subsidies have a significant effect on land-use allocation. An increase in insurance subsidies increases farmland-share, indicating insurance subsidies could be an efficient tool to adjust agricultural land-use allocation.

## 1. Introduction

Land is an important resource for agricultural production and urban development. Generally, land-use change is driven by climate, geographic characteristics, market factors and government policy. Land-use has a significant environmental effect on local regions. Land-use change affects biodiversity, water quality, greenhouse gas (GHG) emission, etc. Searchinger et al. (2008) [1] found the growth of cropland for biofuels increased GHG emission in the U.S. for 167 years. Agricultural land-use also impacts soil-dust emission (Tegen et al., 2004) [2] and climate change (Pielke, 2005) [3]. Besides the environmental effects, agricultural land-use also influences the supply of agricultural products. 

Government policies play an important role in land-use allocation, especially agricultural land-use. For example, an agricultural subsidy on crop production encourages farmers to plant more crops. With the subsidy, crop production brings more net return to farmers, and then farmers attempt to extend the acreage of cropland to achieve higher production. Besides direct subsides, policies on other agricultural products, such as biofuel, will also influence agricultural land-allocation. The extension of biofuels stimulates the demand for ethanol, which makes corn more profitable. As a result, the area where corn is planted will increase. Motamed et al. (2016) [4] found that in the United States, the corn area showed a significant and large response to local ethanol markets.

The objective of this paper is to investigate the effect of crop insurance subsidies on agricultural land-use. In the U.S. scenario, crop insurance is an important instrument for farmers to control the risks from natural disasters and market volatility. The Federal Crop Insurance Corporation (FCIC) was created in 1938, and focused on some major crops in a few regions. In 1980, the government expanded the species and regions covered by federal crop insurance. In order to encourage crop insurance participation, the government improved the crop insurance program, with greater subsidy levels in 1994 and 2000. There are two basic types of crop insurance—yield-based and revenue-based, which guarantee crop production, based on historical yield and price. The Federal Crop Insurance Program helps farmers with risk management. Under the assumption of risk averse, lower risk increases the expected utility from crop-production profit. The higher expected utility of crop-production profit encourages farmers to extend farmland to plant more crops. Schatzki (2003) [5] focuses on the effects of uncertainty and sunk costs on land-use change. He finds that higher uncertainty in returns from potential use will decrease the likelihood of conversion from agriculture to forest. Moreover, crop insurance subsidies reduce the cost of insurance premiums. Lower insurance costs will also increase total profit, encouraging farmers to plant more crops. Therefore, investigating the effect of crop insurance subsidies on agricultural land-use is important: for policy makers, crop insurance subsidies could be considered a tool to adjust agricultural land-use; the change in land-use allocation will influence agricultural-product supply and environment quality in local areas. 

To find the effects of insurance subsidies on agricultural land-use, we apply the multinomial-fractional-logit model with county-level data from all continental states in the U.S. In this paper, we classify land-use type in two ways. The first way is following the classification in Hardie and Parks (1997) [6], which classified farmland into irrigated and unirrigated farmland. The second way is to classify farmland more specifically, such as cropland, woodland, and pastureland. The results show that insurance subsidies increase irrigated/unirrigated farmland, cropland and pastureland allocation. 

The contribution and innovation of this paper is in using the multinomial-fractional- logit model to investigate the effect of insurance subsidies. Based on the single-outcome model, which focused on crop area, discussed in Yu et al. (2018) [7], we innovatively classified land-use into different types. By considering the effects of insurance subsidies on multiple land-uses within the same fractional-response model, we were able to indirectly control for the substitution among various land-use alternatives. 

The paper has five sections. The next section is a literature review, which looks at previous studies in land-use and the effects of government policies. The following part is data description. Then we discuss the theoretical framework and econometric model that would be used. The fourth section contains the results of the estimations of the agricultural census data and the discussion. The final section is the conclusions.

## 2. Materials and Methods

Generally, the usage of land is dependent on the spatial characteristics, demographic characteristics, and land quality. Moreover, land-use is also influenced by market demand and government policy.

Lubowski et al. (2008) [8] focus on the factors that drive land-use change. They analyze change in the U.S. land-use between 1982 and 1997, and consider the net returns as the drivers of land-use change. In their model, the factors from both the supply and demand sides are included. The results show that the private land-use decisions were dependent on land quality, economic returns, and public policies. Veldkamp and Fresco (1996) [9] use a theoretical model to study land-use. They claim that land-use change depends on both biophysical and human demands. Typical biophysical drivers are biophysical suitability, land-use history, and the spatial distribution of infrastructure. Important human land-use drivers are population density, the regional technology-level, economic conditions, attitude and values. Newburn et al. (2005) [10] claim that the site-selection of land-use is influenced by three important factors: biological benefits, land cost, and likelihood of land-use change. After comparing different targeting strategies, they find that the relationship between economics and land-use change is important. Wang et al. (2009) [11] develop a spatial-autoregressive-multinomial-probit model to analyze land-development decisions, including spatial clustering and cross-alternative correlation. The explanatory variables include parcel area, parcel perimeter-to-area ratio, network distances and soil slope. The results show that distance to CBD has a positive effect on the likelihood of residential development. Colsaet et al. (2018) [12] conduct a systematic review on land-take and urban sprawl, and they find population and income growth are important drivers for land-take. Moreover, political and institutional factors such as subsidies for land consumption also determine urban sprawl. 

The research related to the effect of insurance subsidies on land-use is limited. Yu et al. (2018) [7] investigate the effects of crop insurance-premium subsidies on crop acreage. In their study, crop insurance-premium subsidies affect crop acreage in two ways. The first way is by increasing expected returns; the second is by reducing riskiness of crops. These two ways will encourage farmers to increase crop acreage. The results show that a 10% increase in the crop insurance-premium subsidy increases crop acreage by 0.43%. However, Yu et al. (2018) [7] only considered the effect of land-use on crop area, not on other types on farmland. To address the question “why do we subsidize crop insurance?” Barnett and Coble (2012) [13] consider the contributions made by subsidies to the policy objectives. They explain the mechanism behind premium subsidies and outline some potential problems caused by the insurance subsidies. In the end, the authors suggest four research topics, one of which is to study the resource-allocation decisions affected by insurance subsidies. Wu (1999) [14] estimates the effect of crop insurance in the Central Nebraska Basin. He finds that crop insurance would increase chemical use by shifting land from hay and pasture to corn. Goodwin et al. (2004) [15] use multi-equation structural models to analyze the acreage effect of the Federal Crop Insurance Program. The response of crop-acreage change was significant. In some cases, a 30% decrease in premiums, which implied an increase in subsidies, would lead to a crop-acreage increase from 0.2% to 1.1%. Young (2001) [16] investigated the effect of crop insurance on farmers’ planting choices. The simulation results show that crop insurance subsidies had a positive effect on aggregate planting, especially for wheat and cotton. Overall, previous studies on insurance subsidies and land-use focused on the acreage effect on cropland, but the proportion of allocation and effects on other farmland were not mentioned.

There are also many literature reports into other factors that impact agricultural land-use. Prabhakar (2021) [17] claims that in the Asia region, the growing population and related demand are major drivers of land-use change. Paudel et al. (2019) [18] find that in Nepal the population growth is also the main factor of agricultural land-use change. Moreover, transportation, urbanization and government policies also drive the change. Miao (2013) [19] applies a logit land-share model, using panel data from 1997 to 2009. He finds that corn-based ethanol plants have a significant effect on the proportion of land planted with corn. Plantinga et al. (2002) [20] develop a spatial-city model to estimate agricultural land price. In their study, land prices reflect not only current uses of land, but also potential uses. Therefore, the current land value reported is influenced by agricultural production rents and rents from future land development. Mann et al. (2010) [21] focus on the effect of agricultural rent on cropland conversion in the Amazon rainforest. The results show that, besides transportation cost, the expected returns from the venture also affect agricultural expansion. Piquer-Rodriguez et al. (2018) [22] find profit-related factors strongly cause the agricultural intensification in the Pampas, especially for cropland. Lichtenberg (1989) [23] focuses on the effect of land quality on land-use, crop choice, and technological change. He finds that land quality had a significant impact on cropland allocation. Technologies tended to be applied primarily on land of a low quality. Otherwise, the irrigation was sensitive to tax policies.

Hardie and Parks (1997) [6] carry out research on southeastern land-use. They apply an area-based model, and analyze the effect of land quality on the probability of different types of land-use. The variables that are used in this paper are crop revenue, crop cost, saw-timber price, pulpwood price, timber cost, land class, which reflects land quality, population density and per capita income. They also use discrete explanatory variables to find the land-use in different regions. The analysis of this paper is based on the rent-maximization hypothesis, and uses variables such as costs and prices as economic characteristics. However, the authors do not consider the influence of government action, which could also be an important factor affecting land-use allocation.

## 3. Data Source and Variable Selection

The data on land-use acres and agricultural market were collected from the USDA Census of Agriculture. The Census of Agriculture provides county-level data of farmland acreage, land cash-rent, farmers’ net income, and agricultural production expenditures. According to the definition and explanation from the Census of Agriculture, the category of irrigated land includes all land watered by any artificial or controlled means. For approximate land area, the proportion of farmland or cropland area may exceed 100%, since some of the operations have land in two or more counties, while all acres are reported in the principal county of operation. As the multinomial-fractional-logit model that was applied in this paper requires the outcomes to be fractions in the [0, 1] interval, we dropped land shares that exceeded 100%. The land-share data that were applied in the estimation were summed to unity for each county-year, according to construction. Farmland represents the total acres of land in all types of farms. The category of total cropland includes harvested cropland, abandoned cropland, other pasture and grazing-land that could be used for crops without any improvement, and land used for cover crops. Total woodland includes natural or planted woodlots, cutover, deforested land, and pastured woodland. Net cash income is derived by subtracting total farm expenses from total sales, government payments, and other farm-related income. The census data are reported at an interval of every five years. In this paper we use the data from the years 2002, 2007 and 2012, as they are the three most recent reported years. The data contain counties in all continental states.

The data for crop insurance information were collected from the Risk Management Agency (RMA) and Summary of Business (SOB). These data sources provide county-level data of the crop insurance-premium, subsidies from government, coverage level and other information. The data are reported by different species, delivery type, coverage levels and other details. Therefore, we constructed county-level data by summing up subsidy amounts from those subgroups. The average values of crop-market value and subsidies for crop insurance are constructed using the total value and cropland acreage, while the average values of farmer expenses and net income come from dividing the total value by farmland acreage. 

Table 1 shows the summary of the data that were used in the estimation. The category other 1 is calculated by subtracting the irrigated and unirrigated farmland share from one, while other 2 is calculated by subtracting the cropland, woodland and pastureland share from one. From the table, we can find that the average irrigated-farmland share is 1.36%, while the average unirrigated-farmland share is 10.86%. For more detailed land-use types, cropland has the largest average land-share, which is 28.08%, while the pastureland share is 17.82%. The acreage of woodland has the smallest average share, at 6.46%. The average cash-rent is USD 82.70 per acre. The average crop insurance-subsidy is USD 9.048 per acre. In the estimation, all dollar-valued regressors are logged to obtain the effects with percentage change.

## 4. Model

In this paper, the use of land is classified in two ways. The first is following the classification from Hardie and Parks (1997) [6]. Hardie and Parks (1997) [6] claim that irrigation is important. Therefore, we will classify farmland use into irrigated farmland and unirrigated farmland. To investigate the effect of insurance subsidies on more specific land-use, the second classifications of land-use type is cropland, woodland, pastureland, and other land. The profit from a particular land-use depends on its cost and revenue. For example, as the cost of crop production decreases or the revenue from the crop increases, the profit from the crop production increases. With higher profit, land will be more likely to be used for crop production. As the total acreage of each county is constant, the purpose of land-allocation is to maximize total profit from all types of land. Therefore, the share of land in use, *j*, could be represented as a function of independent variables:

*P_j_ = P*(*sub_j_, z_ik_*) (1)

where $p_{j}$ is the proportion of the land-use, *j*. The independent variables should be related to revenue and cost, where $sub_{j}$ represents crop insurance-subsidies of the land-use, *j*, $z_{jk}$ represents other control variables such as farmers’ net income, labor expenses, fertilizer expenses, chemical expenses, cash-rent of land, and some dummy variables. 

We focus on the effect of insurance subsidies, with the hypothesis that insurance subsidies would increase the proportion of farmland. The objective of the Federal Crop Insurance Program is to help farmers reduce the risk from natural disasters and market volatility. Farms that enroll on crop-insurance programs are supposed to have lower risk. Crop insurance subsidies could improve the participation rate; moreover, crop insurance-subsidies also reduce farmer’s cost for the insurance premium, and increase expected returns. With lower risk and higher expected returns, farmers are encouraged to increase crop production. Therefore, marginal land and land with less profitable usage are likely to be transferred into farmland, to achieve high crop-production. Marginal land is the land that is sensitive to the demand of different land-uses. Therefore, the hypothesis of this paper could be raised: the insurance subsidies increase farmland proportion, especially farmland for crop production. The proportion of land with less profitable usage will be decreased.

According to the current and previous research works, the logit/probit model is widely popular for investigating land-use conversion. Based on the theory of maximizing net benefit, Carrion-Flores and Irwin (2004) [24,25] estimated residential land-conversion using the probit model. They used parcel-level data and spatial statistics, and found that urban development was affected by preferences for a lower-density area.

In this paper, we use the multinomial-fractional-logit (MFLOGIT) Model. The MFLOGIT model is used when the outcomes are fractional variables, such as rates and proportions. For example, Mullahy and Robert (2010) [26] applied this model to the time-budget problem. They explored how people with different education levels allocated time to physical activities, where the allocation of time is a sort of fractional outcome. Papke and Wooldridge (1993) [27] introduced the quasi-maximum likelihood estimate (QMLE) to avoid the wrong distribution assumption, leading to a relatively efficient estimation. Based on Papke and Wooldridge (1996) [28], Mullahy (2015) [29] discussed the application of this model on economic-share-data outcomes. He extended the univariate-fractional-regression from Papke and Wooldridge (1996) [28] to the multivariate-fractional-logit model. For the application to land use, Molowny-Horas et al. (2015) [30] applied this model to investigate the effect of natural forces and landscape on land-use. Based on the fractional-regression study by Papke and Wooldridge (1996) [28], they used multivariate data from the Barcelona province, Spain. The results showed that the land-use was not only influenced by geographical and environmental variables, but also credited to the neighboring landscape. 

Following Mullahy (2015) [29], the multinomial logit functional form is

(2) $$E\left[y_{im}|x\right]=\frac{\exp\left(x_{i}\beta_{m}\right)}{\sum_{k=1}^{M}\exp\left(x_{i}\beta_{k}\right)}=\frac{\exp\left(x_{i}\beta_{m}\right)}{1+\sum_{k=2}^{M}\exp\left(x_{i}\beta_{k}\right)},\;m=1,\ldots ,M$$

with normalization $\beta_{1}=0$, where $x_{i}$ represents the independent variables that affect land-use, m represents land-use type, and $y_{im}$ represents share of the mth land-use.

The average partial effects (*APE*) for continuous variables is

(3) $${\hat{APE}}_{mk}=\frac{1}{N}\sum_{i=1}^{N}\frac{\partial E[y_{im}|x_{i}]}{\partial x_{ik}}$$

where

(4) $$\frac{\partial E[y_{im}|x_{i}]}{\partial x_{i}}=\exp\left(x_{i}\beta_{m}\right)*\frac{(1+\sum_{j=2}^{M}\exp\left(x_{i}\beta_{j}\right))*\beta_{mk}-\sum_{j=2}^{M}\exp\left(x_{i}\beta_{j}\right)*\beta_{jk}}{{\left(1+\sum_{j=2}^{M}\exp\left(x_{i}\beta_{j}\right)\right)}^{2}}$$

Consider the potential endogeneity problem, which is that the increase in farmland may cause the increase in total insurance-subsidies; therefore, we use the average subsidies per acre instead of total subsidies. We also use average values per acre for other independent variables, to avoid the endogeneity problem. However, the endogeneity problem still exists, since the increasing farmland-share may decrease the average cash-rent. To avoid this problem further, we estimate the model with all regressors replaced with their lags, as all the explanatory variables are predetermined. 

In the estimation, we will apply the district fixed effect. The agricultural district is classified into nine categories, which are northwest and mountain, north central, northeast, west central, central, east central, southwest, south central, and southeast.

## 5. Results

Table 2 and Table 3 provide the estimated coefficients in the multinomial-fractional-logit model with either the year fixed-effect or the agricultural district fixed-effect, which are shown as Year fe and Dist fe in all tables. The standard errors are clustered at state level. Table 2 shows that the effect of insurance-subsidies on both irrigated land and unirrigated land is significant and positive. The positive coefficients indicate that as premium-subsidies increase, the probability of changing other land into irrigated/unirrigated farmland increases. The effects of average net income on irrigated and unirrigated allocation are not significant. The effects of agricultural-production expenses such as labor expense, fertilizer expense and chemical expense on other land-use are positive and significant, which indicate that as those expenses increase, the land is less likely to be transferred into irrigated and unirrigated farmland.

For the classification of cropland, woodland and pastureland (Table 3), the influence of insurance-subsidies on cropland and pastureland are significant with year fixed- effect. As the amount of insurance subsidies increase, the proportion of cropland and pastureland increases. This result is consistent with the hypothesis, which is the insurance- subsidies from government encourage crop-production, and increase cropland share. However, the effect of insurance-subsidies on woodland land-share is not significant. Average cash-rent per acre has a negative effect on woodland share, but a positive effect on cropland, indicating that as average cash-rent increases, land is more likely to be transferred from woodland into cropland. This result might be due to high profit from cropland. The effect of labor expense on cropland share is negative, but woodland share is positive. As farm-labor expenses increase, landowners may try to abandon some of their agricultural production and transfer cropland into woodland and other land. Labor and fertilizer expenses have a positive effect on other land with both year and district fixed-effects. The increasing agricultural expenses lead to other land-share increases.

Table 4 and Table 5 show the estimated results with lagged variables. The results are similar to the estimation with un-lagged variables, but the significance levels are increased. Crop insurance-subsidies have positive effects on irrigated/unirrigated farmland, cropland and pastureland, while the effects on other land are negative.

Table 6 and Table 7 show the average marginal effect estimated in the multinomial-fractional-logit model with both un-lagged and lagged variables. Signs of coefficients coincide with signs of the corresponding marginal effects. For the effect of insurance-subsidies on irrigated farmland and unirrigated farmland, a 10% increase in average insurance-subsidies per acre increases irrigated farmland-share by 3.127 percentage points, and unirrigated farmland-share by 0.229 percentage points. The marginal effect of insurance-subsidies on unirrigated farmland is smaller than that on irrigated farmland. The marginal effects of average cash-rent and agricultural expenses are also significant. As cash-rent per acre increases by 10%, the unirrigated farmland share increases by 0.245 percentage points, and the other land-share decreases by 0.239 percentage points. Average cash-rent has a positive marginal effect on cropland-share and negative marginal effects on woodland-share. For the effect of labor expense, a 10% increase in expense decreased cropland-share by 1.046 percentage points.

Table 7 shows the estimated average marginal-effects with lagged variables. The marginal effects that are estimated with lags are more reasonable than those without lags. As the crop insurance-subsidies increase by 10%,the shares of irrigated and unirrigated farmland increase by 0.06 and 0.206 percentage points, respectively. With classification 2, lagged average-subsidies increase by 10%, cropland- and pastureland-share increase by 0.265 and 0.292 percentage points, respectively, while woodland-share decreases by 0.05 percentage points. Agricultural expenses such as labor expense, fertilizer expense and chemical expense also have positive average marginal-effects on other land-share under both classifications. The result is consistent with previous studies on crop-acreage effects, which showed insurance-subsidies had a positive effect on crop acreage. Since the effect of insurance-subsidies on both cropland and pastureland is positive, the increased cropland-share may not be transferred from pastureland, but from other types of land. Average cash-rent has a positive marginal-effect on cropland-shares, but a negative effect on woodland-shares. A 10% increase in average cash-rent leads cropland-shares to increase by 1.135 percentage points, and woodland-share to decrease by 0.251 percentage points. It indicates that cropland is likely to have higher cash-rent than woodland, since cropland is more profitable. Production expenditures have a significantly positive marginal-effect on other land-share. As the operation costs increase, farmers are likely to abandon some of their crop production and transfer cropland into woodland and pastureland. 

## 6. Conclusions

This paper investigated the effect of insurance-subsidies on agricultural land-use. As agricultural land-use allocation has great influence on the agricultural market and local environment quality, policy makers should know how policy tools such as subsidies affect land-use allocation. Based on the multinomial-fractional-logit model, which is used with fractional outcomes, we investigate the effect of crop insurance on the allocation of irrigated farmland, unirrigated farmland, cropland, woodland, and pastureland. The data were collected from the Census of Agriculture and Risk Management Agency, from counties in all continental states. To identify the potential endogeneity problem, we used average values and lagged independent-variables in the estimation. The estimated results provided coefficients with both year fixed-effect and agricultural district fixed-effect. The results with lagged variables showed that insurance subsidies, as a kind of government tool, have a significant effect on agricultural land-use allocation. A 10% increase in insurance-subsidies increases the share of irrigated farmland, unirrigated farmland, cropland and pastureland by 0.06, 0.206, 0.265 and 0.292 percentage points, respectively. We can conclude that crop insurance-subsidies have a positive effect on both irrigated farmland-share and unirrigated farmland-share, and that the effect on unirrigated farmland is larger. For more specific classification, as insurance-subsidies increase, lands other than pastureland are likely to be transferred into cropland. The results are consistent with the hypothesis, which claims that crop insurance-subsidies have a positive effect on farmland-allocation, by encouraging farmers to plant more crops.

With regard to policy makers, the objective of crop insurance-subsidies is to help farmers with risk management, which could reduce risk with the guarantee of crop yield or revenue. However, the effect of insurance is not only on production risk, but also on agricultural land-use allocation. As the allocation of agricultural land influences the agricultural market and environment quality, policy makers should consider the effects on land-allocation when they subsidize crop insurance. In addition, crop insurance-subsidies could also be considered as an efficient way for policy makers to adjust agricultural land-allocation. In the future, researchers may investigate more how long the effect of insurance-subsidies lasts on land-use change, so that policy makers could consider this time effect when adjusting land-allocation.

## Figures and Tables

**Table 1:** Data Summary.

Variable	Obs	Mean	Std. Dev.	Min	Max
Irrigated-land share	6872	0.01357	0.02928	0.000003	0.2943
Unirrigated-land share	6872	0.1086	0.08582	0.000017	0.5195
Other 1	6872	0.8778	0.08982	0.4552	0.9997
Cropland share	6872	0.2808	0.2482	0.000689	0.9589
Woodland share	6872	0.06456	0.05253	0.000017	0.3440
Pastureland share	6872	0.1782	0.1985	0.000802	0.9767
Other 2	6872	0.4765	0.2928	0.000114	0.9968
Average subsidy per acre	6131	9.0476	11.8584	0	487.2025
Net income per acre	6863	450.4731	932.9713	−6442.95	22,116.44
Cash-rent per acre	6806	82.6965	141.6212	0.1808	7371.429
Labor expense per acre	6598	24.0726	63.5355	0.04711	1829.218
Fertilizer expense per acre	6856	121.4372	133.5045	0.005908	2657.762
Chemical expense per acre	6795	66.0384	98.3186	0.01042	3489.415

**Table 2:** Estimated Coefficients on Land-Use of Irrigated Farmland and Unirrigated Farmland in Multinomial-Fractional-Logit Model.

	Irrigated Farmland	Unirrigated Farmland	Other 1	Irrigated Farmland	Unirrigated Farmland	Other 1
Average net income	−0.00612	0.0238	−0.0200	0.00334	0.0198	−0.0177
	(0.0558)	(0.0210)	(0.0181)	(0.0527)	(0.0206)	(0.0175)
Average subsidy	0.351 *	0.224 ***	−0.243 ***	0.292 **	0.218 ***	−0.233 ***
	(0.145)	(0.0352)	(0.0326)	(0.103)	(0.0260)	(0.0260)
Average cash-rent	−0.0315	0.231 **	−0.203 **	−0.0253	0.236 **	−0.209 **
	(0.169)	(0.0766)	(0.0668)	(0.168)	(0.0759)	(0.0654)
Average labor expense	−0.110	−0.323 ***	0.299 ***	−0.120	−0.324 ***	0.302 ***
	(0.0808)	(0.0323)	(0.0259)	(0.0833)	(0.0305)	(0.0244)
Average fertilizer expense	−0.524 **	−0.0466	0.121 *	−0.519 **	−0.0772	0.146 **
	(0.175)	(0.0613)	(0.0546)	(0.159)	(0.0609)	(0.0549)
Average chemical expense	0.528 *	−0.242 ***	0.144 **	0.532 **	−0.212 ***	0.119 **
	(0.242)	(0.0614)	(0.0483)	(0.197)	(0.0578)	(0.0452)
_cons	−4.010 ***	−1.679 ***	1.508 ***	−4.081 ***	−1.791 ***	1.621 ***
	(0.285)	(0.179)	(0.159)	(0.340)	(0.193)	(0.186)
Year fe	Yes	Yes	Yes	No	No	No
Dist fe	No	No	No	Yes	Yes	Yes
N	5163	5163	5163	5163	5163	5163

* *p* < 0.1, ** *p* < 0.05, *** *p* < 0.01.

**Table 3:** Estimated Coefficients on Land-Use of Cropland, Woodland and Pastureland in Multinomial-Fractional-Logit Model.

	Cropland	Woodland	Pasture Land	Other 2	Cropland	Woodland	Pasture Land	Other 2
Average net income	0.0539	−0.0120	−0.152 ***	0.0460	0.0380	−0.00703	−0.125 ***	0.0350
	(0.0320)	(0.0281)	(0.0429)	(0.0452)	(0.0275)	(0.0288)	(0.0337)	(0.0371)
Average subsidy	0.216 ***	−0.0502	0.274 ***	−0.299 ***	0.0293	−0.0507	0.302 ***	−0.195 ***
	(0.0416)	(0.0423)	(0.0703)	(0.0613)	(0.0371)	(0.0363)	(0.0599)	(0.0500)
Average cash-rent	0.554 ***	−0.428 ***	−0.135	−0.446 ***	0.568 ***	−0.425 ***	−0.100	−0.451 ***
	(0.132)	(0.0552)	(0.0809)	(0.111)	(0.137)	(0.0525)	(0.0791)	(0.112)
Average labor expense	−0.510 ***	0.137 **	0.0518	0.373 ***	−0.559 ***	0.139 **	0.0404	0.413 ***
	(0.0482)	(0.0451)	(0.0772)	(0.0718)	(0.0434)	(0.0442)	(0.0674)	(0.0632)
Average fertilizer expense	0.318 **	0.514 ***	−0.396 **	0.0361	0.114	0.426 ***	−0.278 *	0.0977
	(0.105)	(0.109)	(0.124)	(0.163)	(0.103)	(0.103)	(0.112)	(0.136)
Average chemical expense	−0.150	−0.140	−0.308 **	0.343 *	0.107	−0.0819	−0.441 ***	0.228 *
	(0.116)	(0.117)	(0.118)	(0.137)	(0.0965)	(0.107)	(0.102)	(0.109)
_cons	−3.016 ***	−2.903 ***	1.726 ***	−0.738	−3.152 ***	−3.044 ***	1.247 ***	−0.243
	(0.282)	(0.277)	(0.310)	(0.428)	(0.287)	(0.271)	(0.287)	(0.360)
Year fe	Yes	Yes	Yes	Yes	No	No	No	No
Dist fe	No	No	No	No	Yes	Yes	Yes	Yes
N	5163	5163	5163	5163	5163	5163	5163	5163

* *p* < 0.1, ** *p* < 0.05, *** *p* < 0.01.

**Table 4:** Estimated Coefficients on Land-Use of Irrigated Farmland and Unirrigated Farmland in Multinomial-Fractional-Logit Model with Lagged Variables.

	Irrigated Farmland	Unirrigated Farmland	Other 1	Irrigated Farmland	Unirrigated Farmland	Other 1
Average net income	−0.0672	0.0174	−0.00592	−0.0717	0.0156	−0.00418
	(0.0507)	(0.0230)	(0.0206)	(0.0492)	(0.0222)	(0.0193)
Average subsidy	0.408 *	0.210 ***	−0.235 ***	0.377 **	0.199 ***	−0.223 ***
	(0.163)	(0.0412)	(0.0390)	(0.124)	(0.0300)	(0.0293)
Average cash-rent	0.0375	0.272 ***	−0.250 ***	0.0348	0.277 ***	−0.254 ***
	(0.146)	(0.0756)	(0.0631)	(0.144)	(0.0761)	(0.0631)
Average labor expense	−0.143 *	−0.289 ***	0.275 ***	−0.146 *	−0.290 ***	0.276 ***
	(0.0705)	(0.0312)	(0.0257)	(0.0695)	(0.0307)	(0.0251)
Average fertilizer expense	−0.466 *	−0.0274	0.0904	−0.501 **	−0.0643	0.126 *
	(0.189)	(0.0652)	(0.0601)	(0.155)	(0.0588)	(0.0531)
Average chemical expense	0.446	−0.288 ***	0.201 ***	0.483 **	−0.257 ***	0.171 ***
	(0.244)	(0.0666)	(0.0529)	(0.178)	(0.0593)	(0.0450)
_cons	−3.834 ***	−1.731 ***	1.536 ***	−3.780 ***	−1.852 ***	1.641 ***
	(0.271)	(0.161)	(0.144)	(0.324)	(0.176)	(0.174)
Year fe	Yes	Yes	Yes	No	No	No
Dist fe	No	No	No	Yes	Yes	Yes
*N*	3106	3106	3106	3106	3106	3106

* *p* < 0.1, ** *p* < 0.05, *** *p* < 0.01.

**Table 5:** Estimated Coefficients on Land-Use of Cropland, Woodland and Pastureland in Multinomial-Fractional-Logit Model with Lagged Variables.

	Cropland	Woodland	Pasture Land	Other 1	Cropland	Woodland	Pasture Land	Other 1
Average net income	0.0336	−0.00681	−0.126 **	−0.00592	0.00538	−0.000495	−0.103 **	−0.00418
	(0.0367)	(0.0305)	(0.0451)	(0.0206)	(0.0329)	(0.0310)	(0.0365)	(0.0193)
Average subsidy	0.261 ***	−0.0765	0.227 **	−0.235 ***	0.139 ***	−0.0819 *	0.236 ***	−0.223 ***
	(0.0522)	(0.0406)	(0.0747)	(0.0390)	(0.0401)	(0.0397)	(0.0669)	(0.0293)
Average cash-rent	0.606 ***	−0.424 ***	−0.125	−0.250 ***	0.629 ***	−0.417 ***	−0.101	−0.254 ***
	(0.130)	(0.0500)	(0.0776)	(0.0631)	(0.135)	(0.0457)	(0.0752)	(0.0631)
Average labor expense	−0.505 ***	0.103 *	0.0542	0.275 ***	−0.519 ***	0.106 *	0.0378	0.276 ***
	(0.0462)	(0.0416)	(0.0710)	(0.0257)	(0.0441)	(0.0417)	(0.0649)	(0.0251)
Average fertilizer expense	0.342 **	0.515 ***	−0.319 **	0.0904	0.126	0.452 ***	−0.224 *	0.126 *
	(0.115)	(0.103)	(0.120)	(0.0601)	(0.102)	(0.0977)	(0.105)	(0.0531)
Average chemical expense	−0.219 *	−0.0826	−0.403 **	0.201 ***	0.0117	−0.0428	−0.503 ***	0.171 ***
	(0.110)	(0.105)	(0.127)	(0.0529)	(0.0920)	(0.0929)	(0.109)	(0.0450)
_cons	−3.110 ***	−3.091 ***	1.542 ***	1.536 ***	−3.110 ***	−3.264 ***	1.019 ***	1.641 ***
	(0.252)	(0.267)	(0.318)	(0.144)	(0.273)	(0.272)	(0.287)	(0.174)
Year fe	Yes	Yes	Yes	Yes	No	No	No	No
Dist fe	No	No	No	No	Yes	Yes	Yes	Yes
*N*	3106	3106	3106	3106	3106	3106	3106	3106

* *p* < 0.1, ** *p* < 0.05, *** *p* < 0.01.

**Table 6:** Estimated Marginal Effects on Land-Use in Multinomial-Fractional-Logit Model.

	Classification 1			Classification 2			
	Irrigated Farmland	Unirrigated Farmland	Other 1	Cropland	Woodland	Pasture Land	Other 2
Average net income	−0.0017	0.0019	−0.0019	0.0064	−0.0009	−0.0174 ***	0.0114
	(0.0559)	(0.0021)	(0.0021)	(0.0055)	(0.0017)	(0.0054)	(0.0097)
Average subsidy	0.3127 ***	0.0229 ***	−0.0275 ***	0.0086	−0.0034 *	0.0397 ***	−0.0490 ***
	(0.0944)	(0.0029)	(0.0031)	(0.0069)	(0.0020)	(0.0082)	(0.0115)
Average cash-rent	−0.0343	0.0245 ***	−0.0239 ***	0.1054 ***	−0.0251 ***	−0.0136	−0.0943 ***
	(0.1691)	(0.0079)	(0.0075)	(0.0247)	(0.0038)	(0.0094)	(0.0250)
Average labor expense	−0.1196	−0.0340 ***	0.0351 ***	−0.1046 ***	0.0080 ***	0.0081	0.0865 ***
	(0.0860)	(0.0031)	(0.0029)	(0.0072)	(0.0024)	(0.0092)	(0.0138)
Average fertilizer expense	−0.5434 ***	−0.0066	0.0159 **	0.0187	0.0285 ***	−0.0400 ***	0.0293
	(0.1513)	(0.0065)	(0.0065)	(0.0204)	(0.0061)	(0.0135)	(0.0322)
Average chemical expense	0.5553 ***	−0.0234 ***	0.0147 ***	0.0207	−0.0066	−0.0490 ***	0.0477 *
	(0.1939)	(0.0060)	(0.0052)	(0.0191)	(0.0066)	(0.0129)	(0.0254)

* *p* < 0.1, ** *p* < 0.05, *** *p* < 0.01.

**Table 7:** Estimated Marginal Effects on Land-Use in Multinomial-Fractional-Logit Model with Lagged Variables.

	Classification 1			Classification 2			
	Irrigated farmland	Unirrigated Farmland	Other 1	Cropland	Woodland	Pasture Land	Other 1
Average net income	−0.0011	0.0014	−0.0003	0.0009	−0.0005	−0.0136 ***	−0.0003
	(0.0008)	(0.0023)	(0.0023)	(0.0062)	(0.0018)	(0.0053)	(0.0023)
Average subsidy	0.0060 **	0.0206 ***	−0.0257 ***	0.0265 ***	−0.0050 **	0.0292 ***	−0.0257 ***
	(0.0026)	(0.0033)	(0.0036)	(0.0073)	(0.0022)	(0.0081)	(0.0036)
Average cash-rent	0.0006	0.0284 ***	−0.0289 ***	0.1135 ***	−0.0251 ***	−0.0131	−0.0289 ***
	(0.0023)	(0.0078)	(0.007)	(0.0231)	(0.0033)	(0.0088)	(0.007)
Average labor expense	−0.0023 *	−0.0305 ***	0.0321 ***	−0.0953 ***	0.0060 ***	0.0066	0.0321 ***
	(0.0014)	(0.0031)	(0.0029)	(0.007)	(0.0023)	(0.0082)	(0.0029)
Average fertilizer expense	−0.0079 ***	−0.005	0.0129 **	0.0237	0.0291 ***	−0.0310 ***	0.0129 **
	(0.0023)	(0.0062)	(0.0061)	(0.0201)	(0.0056)	(0.012)	(0.0061)
Average chemical expense	0.0077 ***	−0.0275 ***	0.0203 ***	0.0018	−0.0037	−0.0532 ***	0.0203 ***
	(0.0024)	(0.0061)	(0.0052)	(0.0174)	(0.0056)	(0.013)	(0.0052)

* *p* < 0.1, ** *p* < 0.05, *** *p* < 0.01.

## Data Availability

All sample data sets are downloaded from the website. Data are available at https://www.nass.usda.gov/AgCensus//, https://www.rma.usda.gov/, accessed on 1 January 2022.
